# Supplementary figures and images for: Components of iron–Sulfur cluster assembly machineries are robust phylogenetic markers to trace the origin of mitochondria and plastids
Source: PLoS Biol. 2023 Nov 8;21(11):e3002374. doi: 10.1371/journal.pbio.3002374 (PMC10631705; doi:10.1371/journal.pbio.3002374)

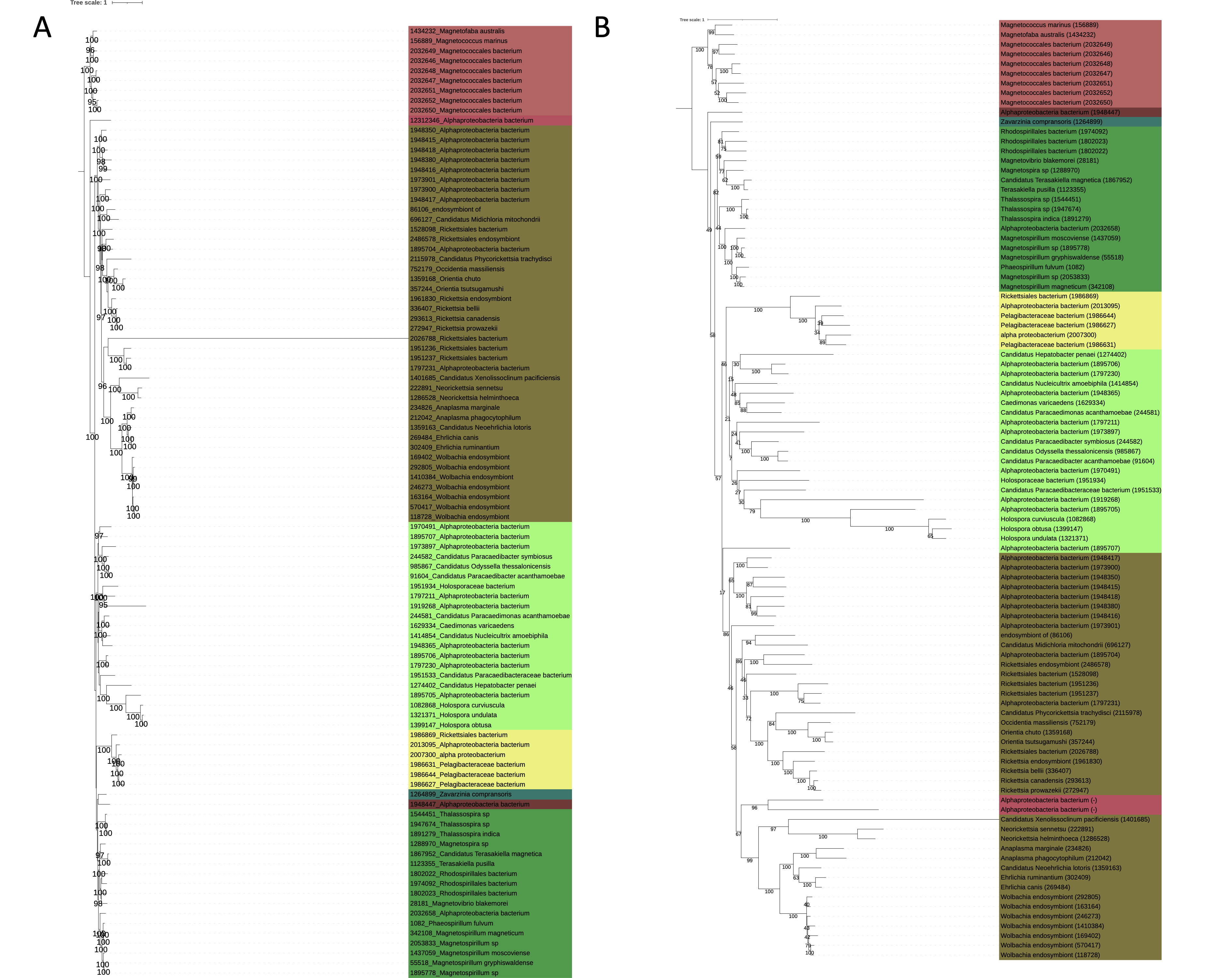

Supplement: S1 Fig — (A) Reference tree of Alphaproteobacteria possessing an ISC system (IF2+RpoB+RpoC). IQ-TREE, LG+F+R7+C60+PMSF, 3,483 amino acid positions, 96 sequences. Numbers at branches indicate ultrafast-bootstrap values. The scale bar indicates average number of substitutions per site. Numbers at the tips indicate taxonomy IDs from NCBI. (B) ML Phylogeny of alphaproteobacterial ISC (IscA+IscS+HscA+Fdx+CyaY). IQ-TREE, LG+R7+C60+PMSF, 1,275 amino acid positions, 97 sequences. Numbers at branches indicate nonparametric bootstrap values. The scale bar indicates average number of substitutions per site. Numbers at the tips indicate taxonomy IDs from NCBI. The data underlying this Figure can be found in S1 Data. (TIFF) [file pbio.3002374.s001.tiff]

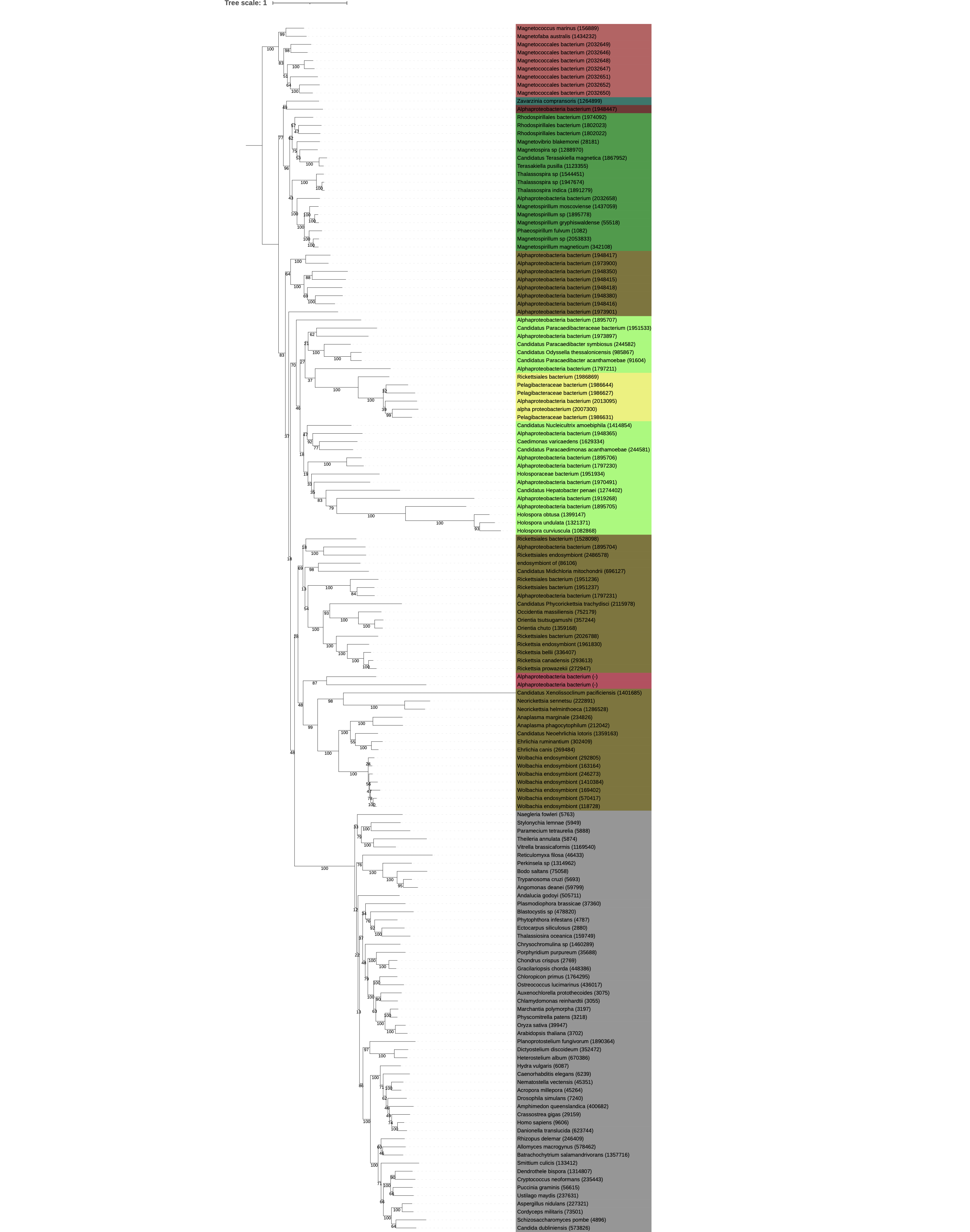

Supplement: S2 Fig — IQ-TREE, LG+R8+C60+PMSF, 1,306 amino acid positions, 149 sequences. Numbers at branches indicate nonparametric bootstrap values. The scale bar indicates average number of substitutions per site. Numbers at the tips indicate taxonomy IDs from NCBI. The data underlying this Figure can be found in S1 Data. (TIFF) [file pbio.3002374.s002.tiff]

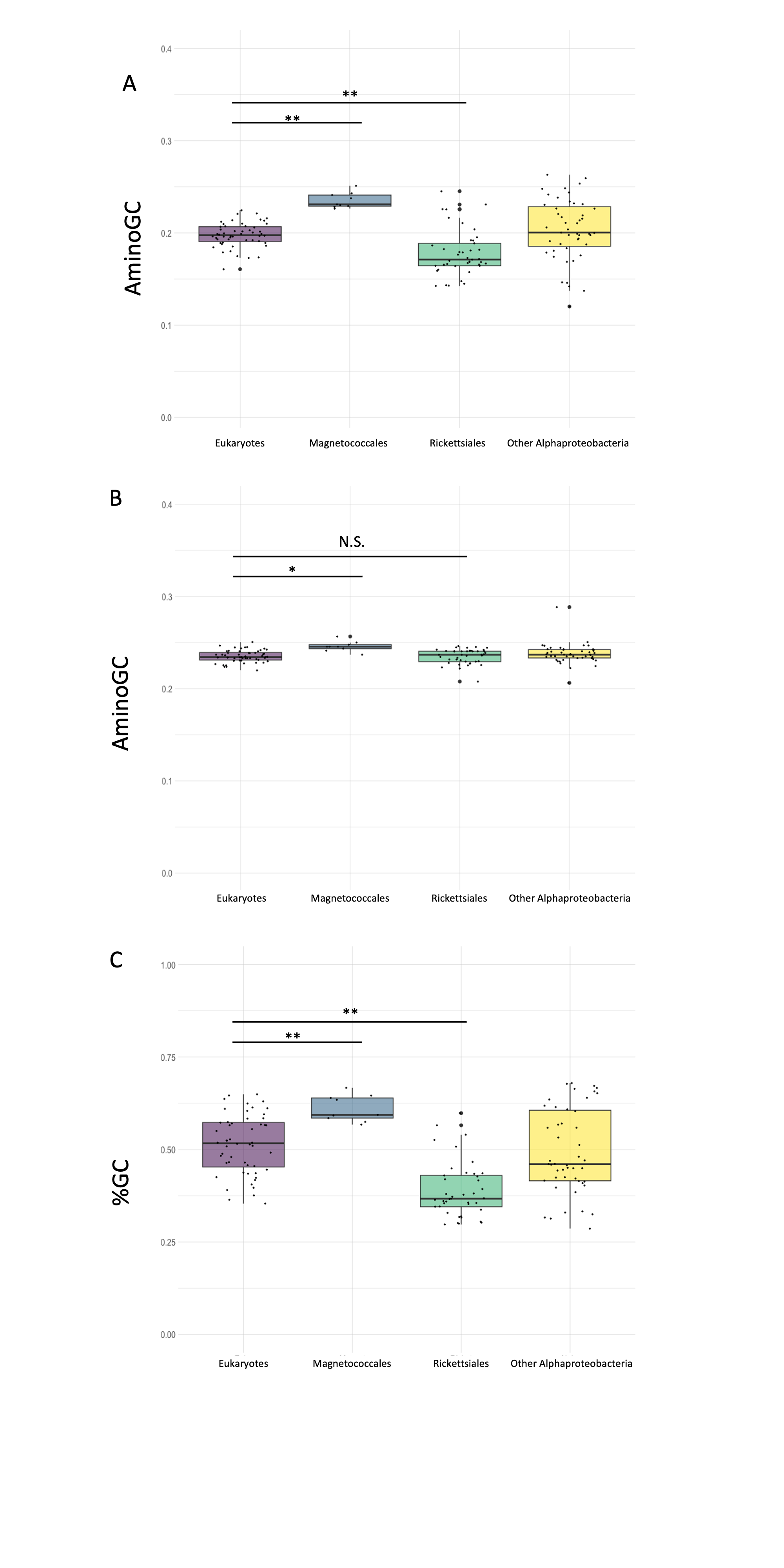

Supplement: S3 Fig — (A) AminoGC for protein concatenation. (B) AminoGC for protein concatenation after stationary-based trimming. (C) %GC for nucleic protein concatenation. The AminoGC was calculated by the script prune_ali.pl from Martijn and colleagues [4]. Middle bar: median; hinges: first and third quartiles; whiskers: largest value no further than 1.5 (interquartile range); dots: individual points; big dots: outliers. The upper bars correspond to the two-tailed Mann–Whitney U test result (H0 = no difference between the 2 groups). N.S.: p > 0.05, *: p < 1 × 10^−3^, **: p < 1 × 10^−5^. (TIFF) [file pbio.3002374.s003.tiff]

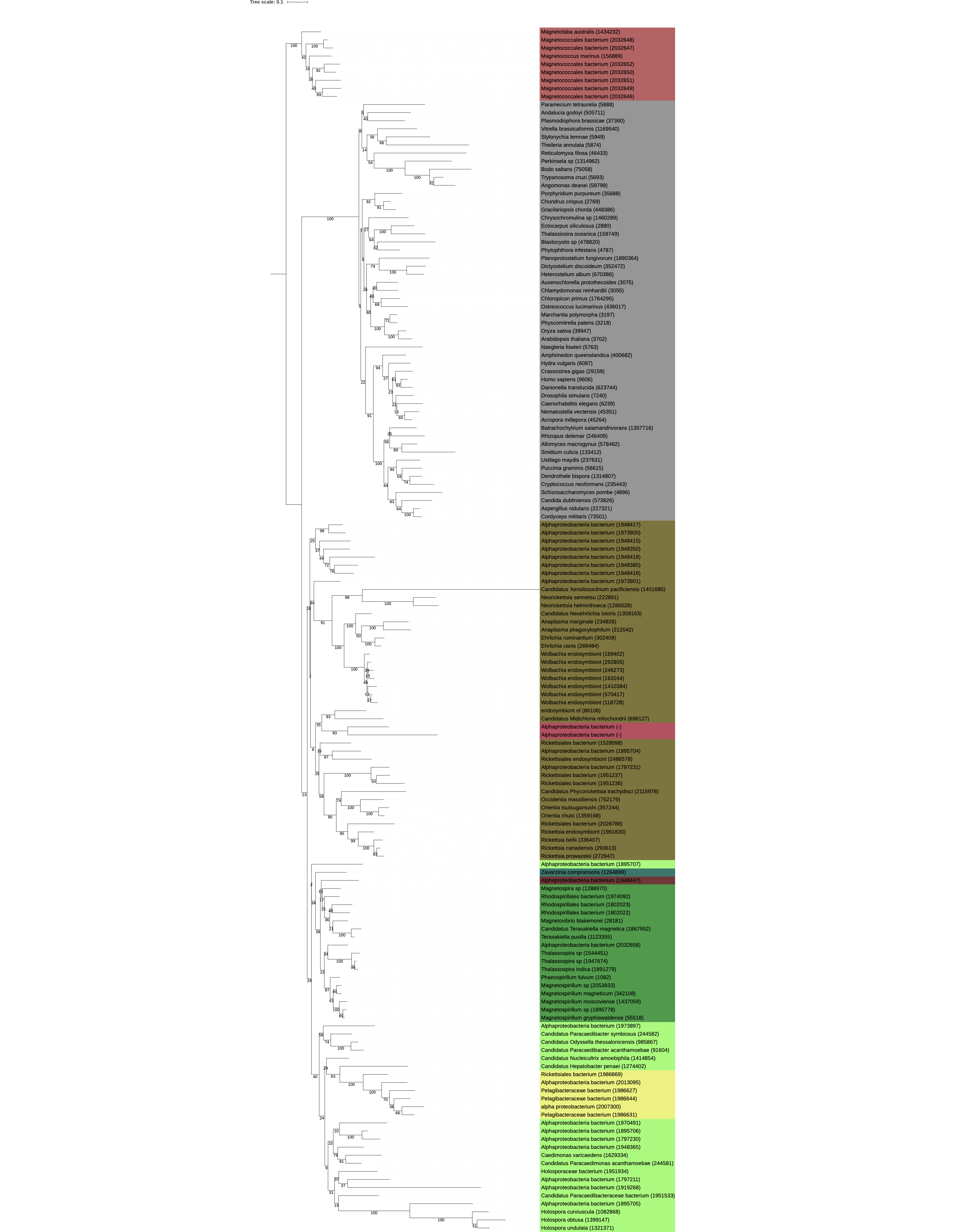

Supplement: S4 Fig — IQ-TREE, LG+R6+C60+PMSF, 532 amino acid positions, 149 sequences. Numbers at branches indicate the nonparametric bootstrap values. The scale bar indicates average number of substitutions per site. Numbers at the tips indicate taxonomy IDs from NCBI. The data underlying this Figure can be found in S1 Data. (TIFF) [file pbio.3002374.s004.tiff]

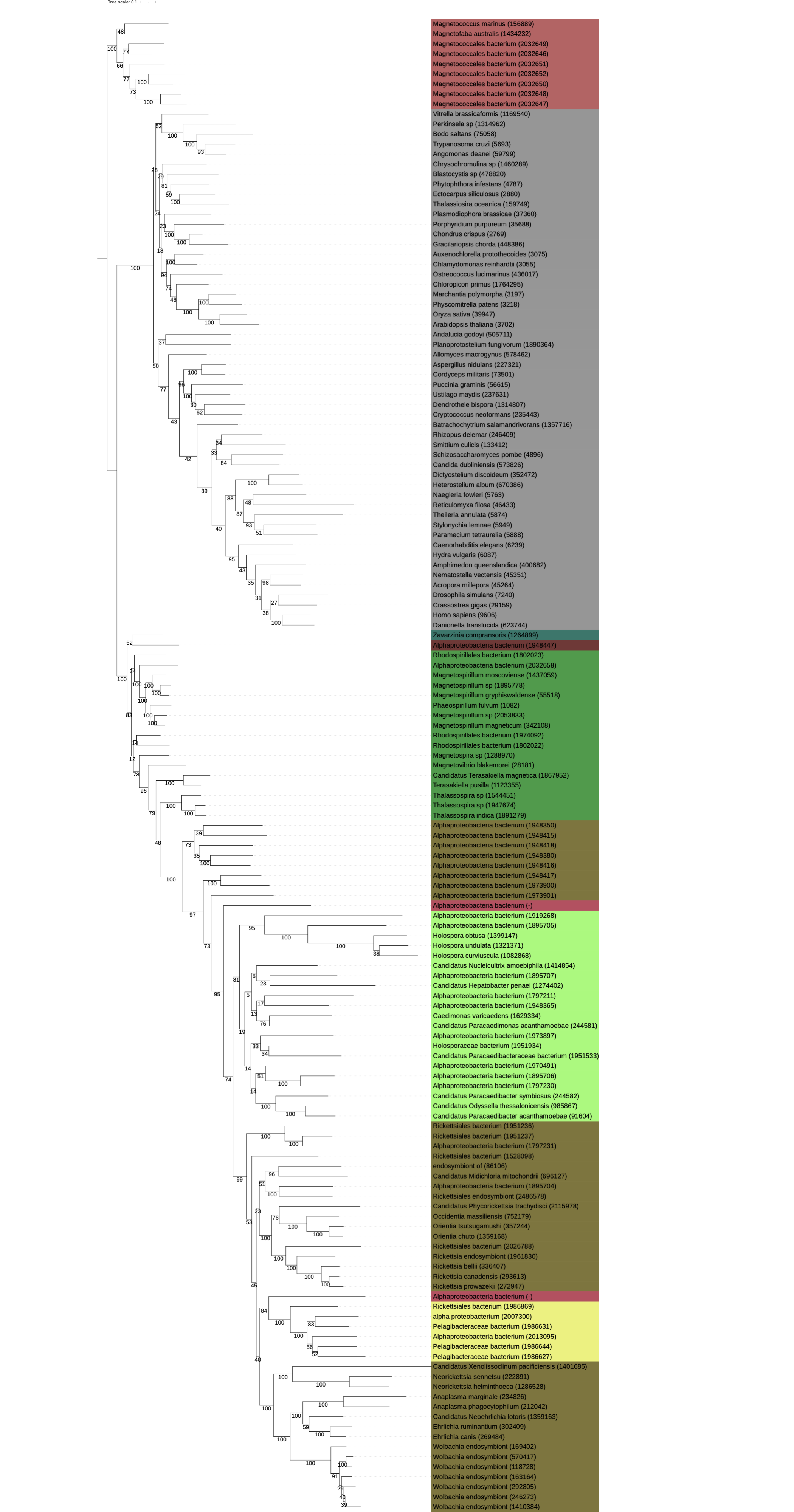

Supplement: S5 Fig — IQ-TREE, GTR+F+R8, 4,128 nucleic positions, 149 sequences. Numbers at the branches indicate nonparametric bootstrap values. The scale bar indicates average number of substitutions per site. Numbers at the tips indicate taxonomy IDs from NCBI. The data underlying this Figure can be found in S1 Data. (TIFF) [file pbio.3002374.s005.tiff]

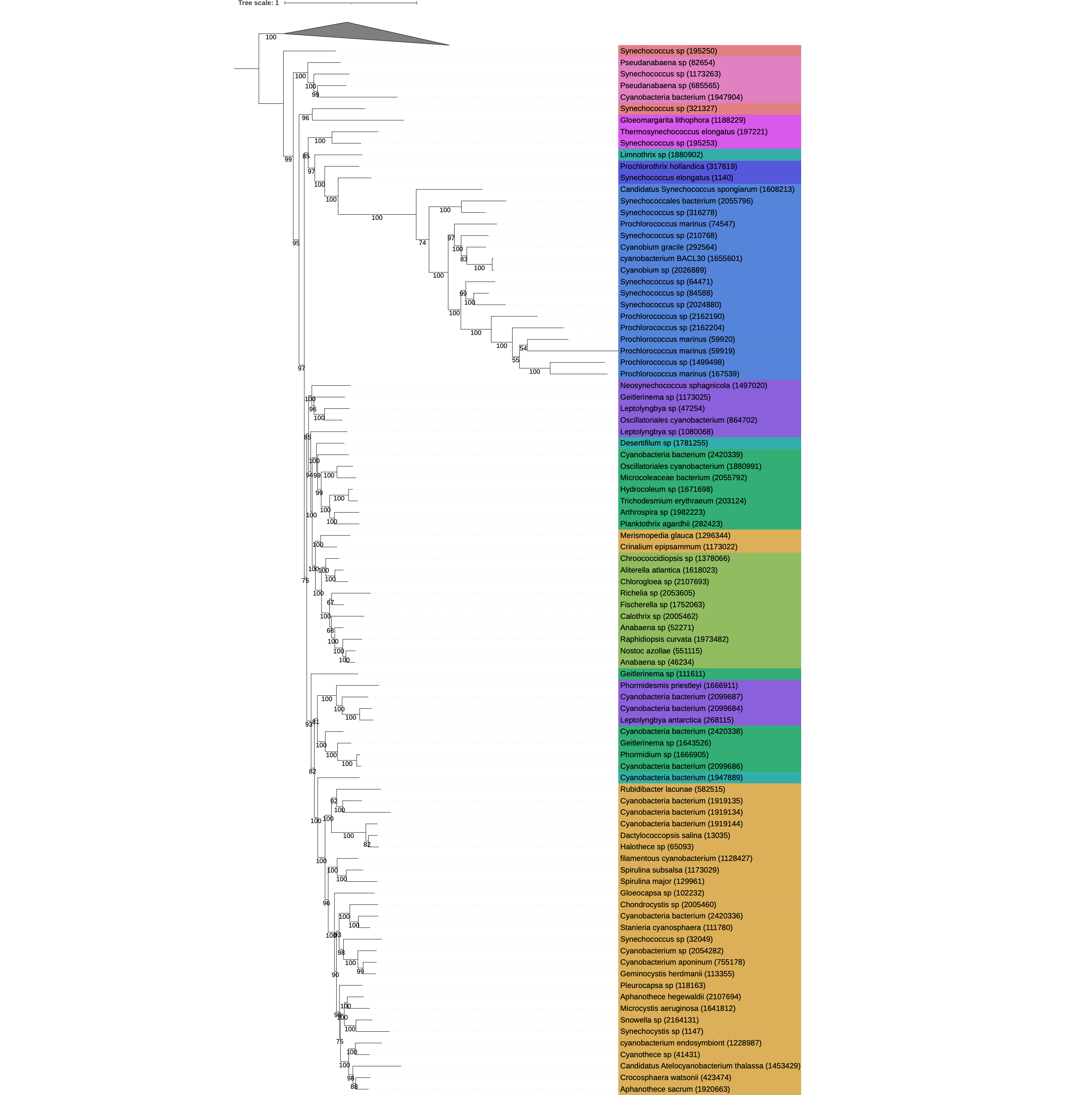

Supplement: S6 Fig — IQ-TREE, LG+F+R10+C60+PMSF. 1,561 amino acid positions, 167 sequences. The numbers at the branches indicate the ultrafast-bootstrap values. The scale bar indicates the average number of substitutions per site. Numbers at the tips indicate taxonomy IDs from NCBI. The data underlying this Figure can be found in S1 Data. (TIFF) [file pbio.3002374.s006.tiff]

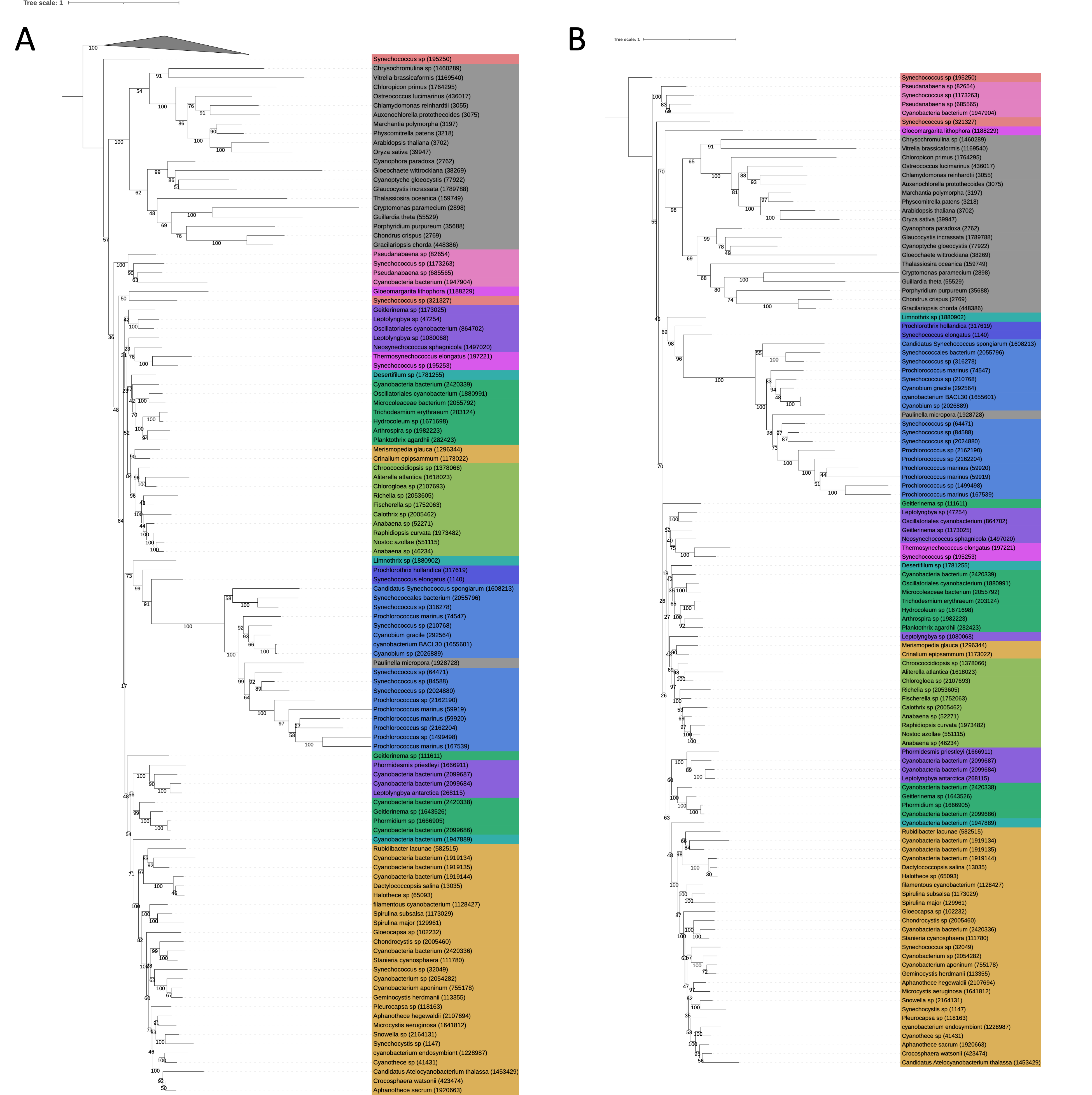

Supplement: S7 Fig — (A) ML phylogeny of cyanobacterial and eukaryote SUF rooted with an external group (without stationary-based trimming). IQ-TREE, LG+R10+C60+PMSF. 1,553 amino acid positions, 188 sequences. The numbers at the branches indicate the nonparametric bootstrap values. The scale bar indicates the average number of substitutions per site. Numbers at the tips indicate taxonomy IDs from NCBI. (B) ML phylogeny of cyanobacterial and eukaryote SUF (without stationary-based trimming). IQ-TREE, LG+R8+C60+PMSF. SufB+SufC+SufD+SufS. 1,565 amino acid positions, 112 sequences. The numbers at the branches indicate the nonparametric bootstrap values. The scale bar indicates the average number of substitutions per site. Numbers at the tips indicate taxonomy IDs from NCBI. The data underlying this Figure can be found in S1 Data. (TIFF) [file pbio.3002374.s007.tiff]

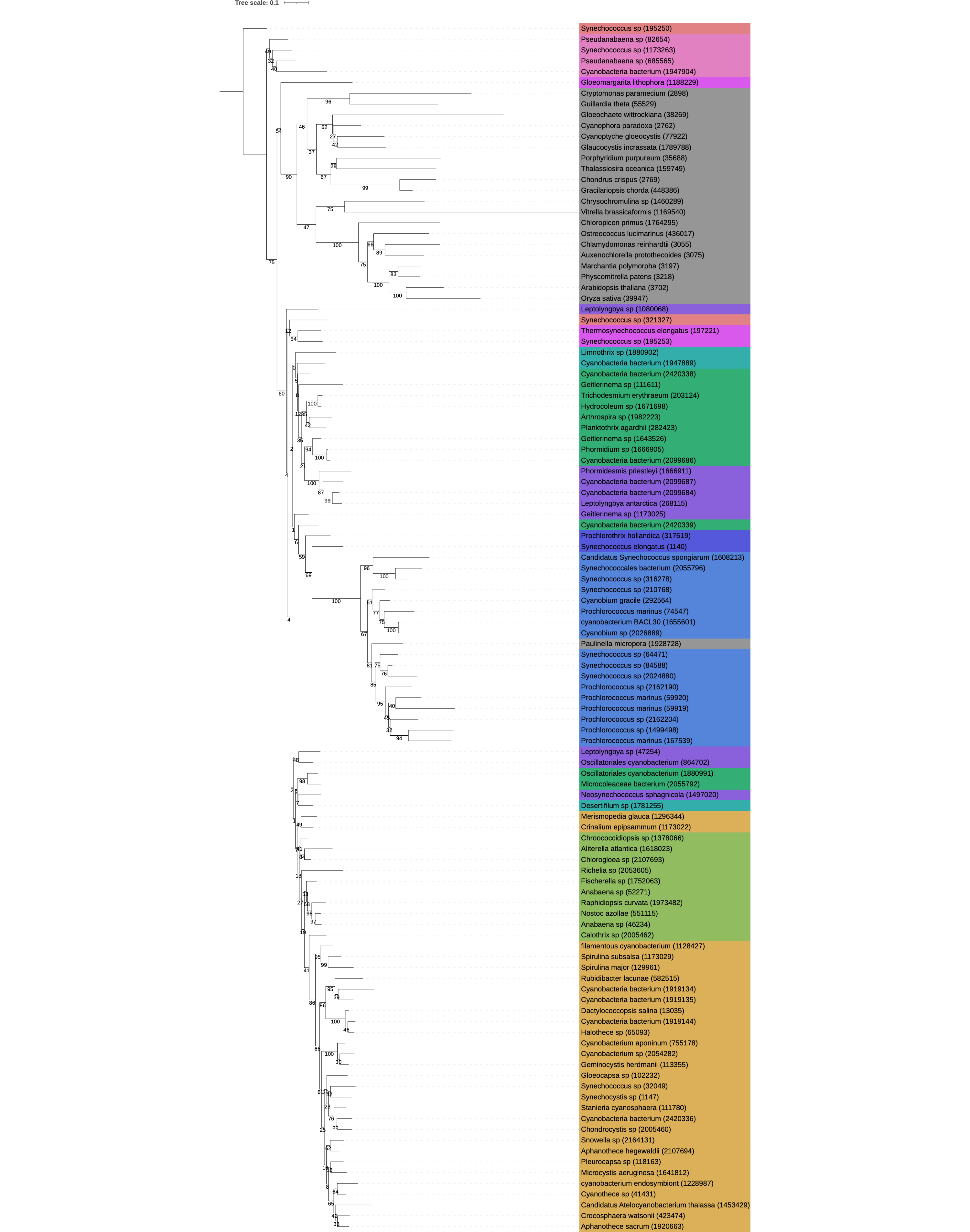

Supplement: S8 Fig — IQ-TREE, LG+R6+C60+PMSF. SufB+SufC+SufD+SufS. 813 amino acid positions, 112 sequences. The numbers at the branches indicate the nonparametric bootstrap values. The scale bar indicates the average number of substitutions per site. Numbers at the tips indicate taxonomy IDs from NCBI. The data underlying this Figure can be found in S1 Data. (TIFF) [file pbio.3002374.s008.tiff]

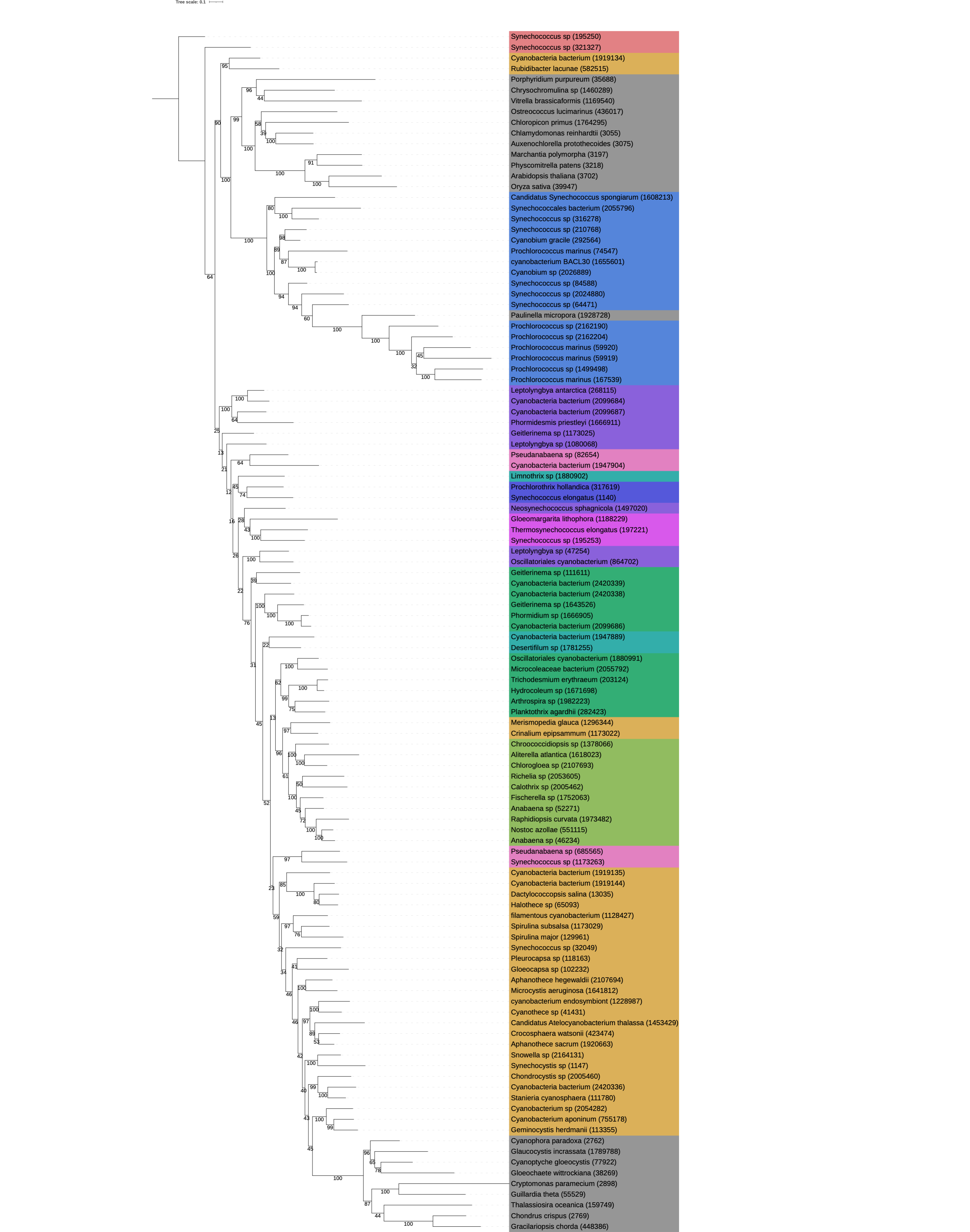

Supplement: S9 Fig — IQ-TREE, SYM+R8. SufB+SufC+SufD+SufS. 4,809 nucleic positions, 112 sequences. The numbers at the branches indicate the nonparametric bootstrap values. The scale bar indicates the average number of substitutions per site. Numbers at the tips indicate taxonomy IDs from NCBI. The data underlying this Figure can be found in S1 Data. (TIFF) [file pbio.3002374.s009.tiff]
