# Supplementary material for: Components of iron–Sulfur cluster assembly machineries are robust phylogenetic markers to trace the origin of mitochondria and plastids
Source: PLoS Biol. 2023 Nov 8;21(11):e3002374. doi: 10.1371/journal.pbio.3002374 (PMC10631705; doi:10.1371/journal.pbio.3002374)
Supplement: Attachment — Submitted filename: Answer-to-reviewers.docx [file pbio.3002374.s019.docx]

Reviewer #1:

The authors present an analysis of iron-sulfur cluster biosynthesis genes to look at the endosymbiotic origin of plastids and mitochondria. The evolution of these organelles has been controversial pretty much forever - their endosymbiotic origin is now beyond any serious question, but this itself has raised new questions and they are also sometimes tied to debates about the origin of eukaryotes as a whole. Any new insights on this subject are potentially important and significant, so evolutionary biologists have been attacking this from several directions, mostly using genomics. One approach of late has been to use nucleus-encoded genes for proteins targeted to the organelles to assess their origins, since organelle genomes may be more direct, but their sequence evolution has been odd and confounding. Here, the authors use a pathway share by both organelles, but using different non-homologous versions of the pathway, to see if it sheds any light on their origin. This does not actually touch on some of the more hotly debated topics (e.g. the whole "mitochondrion-early vs -late debate that seems to never end), but it is useful as the results seems relatively solid and they do touch on other debates such as the nearest bacterial relatives of these organelles. The results here are not surprising in that they seem to support existing hypotheses, but I think this should not be a strike against the work as we do tend to favour "surprising" and likely wrong things over actually supporting something that is less surprising but real in this field. The main results of interest to my mind are the position of the mitochondria deep and not with the rickettsiales (the traditional placement, but one that was always potentially a long-branch or compositional bias artifact). This is not the first paper to suggest this, but this is a very big shift should this come to be the favoured explanation of the data, so it is quite important. The paper is short, and

this is a suitable length since they authors explain what needs explaining for the most part. I have a few suggestions however, some of which are pretty important I think. These are in no particular order, so I will try to note the most important.

1. I think the main problem i have is the lack of glaucophytes in the plastid analysis. These are one of only three primary plastid groups, and the order of those three is hotly contested. Why are they absent? Is there a reason, if so I missed it. If not, I am sorry to say I think they really need to be added and their relationship with the reds and greens noted. This might even prove to be an important point depending on what the analyses say. Also at least need to see if they change anything else. Overall the taxon selection is pretty low considering all the data around these days (from the plastid-containing eukaryotes, the taxon selection in the bacteria is much better).

Our main goal was not to study the internal relationship among photosynthetic eukaryotes but only the origin of the primary plastids. However, we agree with the referee that increased taxonomic sampling can be interesting, also to strengthen tree reconstruction.

In several lineages, SufBC are encoded in the plastid genomes, and SufDS in the nucleus (this information is now included in the large excel sup table 4 showing the taxonomic distribution).

We did not have any Glaucophyta because our initial database originates from NCBI in which only one assembly of the genome is available (*Cyanophora paradoxa*), with low quality and not annotated. We have now added to our database four plastid genomes from Glaucophyta, were we identified SufB and SufC homologues (SufD and SufS being nuclear encoded). We also added two more Rhodophyta nuclear encoded SufD and SufS to enrich the basal lineages (the plastid genome is not available for these assemblies).

We added these sequences in the concatenation and rerun the entire SUF analysis. The addition of these lineages greatly stabilizes the position of plastids within the Cyanobacteria tree, sister to *Gloeomargarita* (see new Figure 2).

Concerning the relative order of the three primary photosynthetic lineages, we find the order (Green, (Glaucophyta,Red)). However, we think that more data is necessary to support this claim and we prefer not to insist on this aspect as the internal phylogeny of eukaryotes is not the main focus of our paper.

2. The SUF/ISC distribution in alphaproteobacteria is interesting. The contention here is clearly that ISC is ancestral and was replaced repeatedly by SUF. Is this repeated replacement true in other groups? Is this something that is already observed and tested? It seems like an important point that needs slightly more discussion and references if it is already discussed elsewhere.

Indeed, we previously observed this pattern in several bacteria and especially in Proteobacteria (Garcia et al. 2022), We discussed that both SUF and ISC were present in the ancestor of Proteobacteria and only one system was maintained in most lineages (so no replacement), either by one or multiple losses, potentially followed by HGT. In the current analysis, what is most important is the distribution of ISC and SUF in the Alphaproteobacteria. We have now added information about the presence of similar patterns to the text and refer to Garcia NEE.

3. The point that "although another study has suggested that they branch with a more distal group11" needs to be more specific. Please spell out where they went in this analysis.

Following the advice of reviewer #3 and given the results of more recent analyses, we have removed the sentence and the associated reference.

4. In the figures (part D of both), what are the numbers on the taxon names? It looks kind of "raw". I suggest the authors should take a bit more time to clean up the figure to make it easier for the non-expert to read. Get rid of internal numbering and underscores etc that mean nothing to the reader. The figure looks like "direct from analysis", and not a finished figure. I always think the reader deserves a bit of work to make the data easy to grasp.

These numbers correspond to NCBI taxID, and this is now specified in the legends. We think that displaying Taxonomic IDs is crucial for the readership to quickly find what genome it corresponds to, so we prefer to keep it in parentheses.

5. (Unfortunately the pages are not numbered so I am going from the PDF off the web site) On page 13 and 14 the authors note they did "1,000 fast-bootstrap replicates" with IQ-tree. these are something specific to this program and need to be interpreted differently from "normal" bootstraps so this should be noted (they are inflated). You can run non-parametric bootstraps with PMSF, and they are not as inflated, so that is worth considering.

The referee is correct. We have rerun the analyses with non-parametric bootstraps and changed the Figures and M&M accordingly.

6. On page 14 the Bayesian analyses are described based on two chains, what happened with the other chains? Did they all converge? It is important if they don't to say what they did.

In both analyses, we made the consensus tree with the two chains that converged the most. For ISC, the other maxdiff results correspond to lower but acceptable convergence (0.23, 0.25, 0.22, 0.32, 0.32). In contrast, for SUF, the rest of the other maxdiff results correspond to non-convergent runs (0.75, 0.45, 0.87, 0.58, 0.60). This is indeed interesting and probably related to the lowest resolution of the SUF analysis. We now detail this in the M&M section.

Reviewer #2:

The paper by Garcia et al deals with the origin of the Iron-sulfur cluster machineries at the onset of mitochondria and plastids. In this paper, the authors extended their previous prokaryotic analysis regarding the different iron sulfur clusters biosynthesis pathways to eukaryotes and, based on a careful phylogenetic analysis of the core ISC and SUF components, have retrieved a deep emergence of mitochondria after the divergence of Magnetococcales and plastids, as a sister clade of Cyanobacteria.

As the authors point out, in the last years there has been several papers addressing the origin of mitochondria (and plastids) with contradictory results. We should keep in mind that phylogenetic analysis are a proxy for likelihoods and not certainties. Moreover, they are highly dependent on the methods and datasets used. I doubt that this paper will solve the current literature debate, but will surely contribute to the discussion.

Having said that, this is a timely paper, that uses nuclear encoded genes as proxy for the origin of mitochondria and plastids. The analyses presented in here are careful and all data to retrieve the authors steps is provided in supplementary information. As also observed by the authors, different methods, can give raise to different results.

I have only some suggestions with the aim to clarify and further support the authors methodology.

- It would be relevant, to present as supplementary figures and/or in newick/figtree format, the preliminary single gene phylogenies containing also the paralogous that were excluded, as well as the non-monophyletic behaviour of some of the proteins (IscU, HscB). This will give strength to the methodology used.

We provided in the Supplementary data the single gene trees corresponding to Prokaryote and Prokaryote+Eukaryote inferred with fasttree, in figtree format, annotated with colors corresponding to the groups of interest (Alphaproteobacteria, Cyanobacteria, Eukaryotes). These trees include all cases of non-monophyly and the paralogues which were excluded from the concatenation. We have now added a file with the color correspondence and information in the main text and M&M.

- An UFB support of 35% is meaningless. According to the IQTree authors, UFB below 95% are not significant (at least for the current version). Even if in the sentence regarding the stationary-based method trimming ML phylogeny, reference to the poor support is given, this could perhaps be rephrased to indicate that in this phylogeny, the branching of eukaryotes is not clear (and not start the sentence with In contrast…)

We rerun the analyses using non-parametric bootstrap. We indicate in the text when there is low or very low support. We removed "In contrast".

Reviewer #3:

In this work, Garcia and colleagues attempt to use two sets of proteins belong to the iron-sulfur cluster machineries ISC and SUF, to infer the phylogenetic origin of the mitochondrion and plastid, respectively.

While the methods are sound, there are profound issues with the interpretation of the results and their novelty in comparison to what is already known.

--- Misrepresentation of the state-of-the-art ---

The authors exaggerate the real existence of a debate when it comes to the origin of mitochondria and plastids. While mitochondrial origins WERE debated for a long time, the two most sophisticated analyses on these questions in terms of complex phylogenetic approaches and broad taxon sampling (Martjin, Nature 2018; Munoz-Gomez NEE 2022) both agree on the deep phylogenetic placement of mitochondria and have given strong methodological arguments for the invalidity of the results presented elsewhere.

We absolutely agree with the reviewer and value greatly these two analyses. However, we cannot ignore these previous works, in particular the last exchange that occurred between the groups of Ettema and Martin in NEE 2022. As underlined by the two other referees, we think our analysis adds significant support to the Martijn and Munoz Gomez studies and is presented as such. We have nevertheless now modified the abstract and introduction to emphasize that we confirm these latest analyses with a new dataset.

When it comes to plastids, since the discovery of Gloeomargarita (Ponce-Toledo 2016), there has been NO conflicting reports about their status as the closest relatives to plastids. Their relationship to plastids is even easily recovered and supported in 16S+23S phylogenies. Any "debate" that might have existed has been put to rest since 2016.

Again, for a matter of honesty, we wanted to mention all placements published recently and not selectively ignore some. We agree that the Ponce-Toledo analysis is the latest one that has not yet been challenged, and we have decided to remove the earlier reference of Ochoa De Alda 2014 as it is not relevant anymore. We changed the text accordingly.

In addition, the writing about what is known on the origin of the SUF and ISC eukaryotic machineries is somewhat unclear. It has long been reported that they are of plastid and mitochondrial origin, respectively. However, when the authors report their results here, they fail to make it clear that there was no doubt about this. For example "Among eukaryotes, SUF homologues are present only in photosynthetic lineages, and preliminary single gene tree analyses place them with Cyanobacteria, confirming their plastid origin." This has long been known, and reported in one of the reviews cited here and references therein (Tsaousis 2019).

We agree that this has been common knowledge for a long time in the community of protistologists. However, analyses in the literature are scattered and mostly based on single markers and no deep phylogenomic approaches. We have nevertheless clarified the text and added a number of references.

Even the distribution of the ISC and SUF proteins in Alphaproteo and Cyanobacteria has been reported elsewhere by the same authors (Garcia NEE 2022) and yet here, the authors present it as if it were new. E.g. "We then used the same approach to investigate the origin of plastids by analyzing the SUF system. 95 genomes covering the current diversity of Cyanobacteria were scrutinized. Whereas the SUF system is largely present in most genomes, the ISC system is practically absent."

We agree that this sentence was misleading and clarified this point in the main text.

--- Ad hoc arguments against previously used phylogenetic markers ---

The authors state that "These previous studies relied on the use of large concatenations of protein markers involved in various cellular processes and encoded either in the nucleus or the organelle, which likely drives different evolutionary constraints that may lead to phylogenetic instability." without providing further proof that this is the case and simply use this as a reason for why there is a need for other phylogenetic markers (i.e. theirs).

We never claimed that our markers fit better as compared to the former ones. We used a single system that corresponds to an essential function that has not yet been used for such purpose. We meant this as a complementary dataset and not to say that the previous analyses were bad. However, for the sake of simplicity, we have removed the sentence.

--- Arguments for the using the SUF and ISC proteins as "better phylogenetic markers" are flawed ---

At the end of this paper, the authors summarize the four reasons for using the SUF and ISC protein to interrogate the origin of plastids and mitochondria, and most can be refuted:

1) "they carry out essential functions and are taxonomically well distributed":

While this is true in eukaryotes, the ISC system is absent from large alphaproteobacterial clades, as seen in Fig 1 and in the text "ISC being present mostly in the basal lineages (Magnetococcales, Marine Proteobacteria 1, MC1) as well as in Rickettsiales, while it is absent in most of the other Alphaproteobacteria,". It seems to be a very odd system to use to place eukaryotes relative to alphaproteobacterial then. How would you be able to infer a more nested position of mitochondria within alphaproteobacterial if most lineages don't possess homologues of the studied system? That strongly discredit the validity of such proteins to address this evolutionary question.

We do not understand the argument. How could eukaryotic ISC arise from a lineage that does not have it? The only possibility is that it lost it after mitochondrial endosymbiosis, but this seems an ad hoc explanation. In any case, we certainly do not use this as the sole argument to infer a deep origin, but rather as an extra hint at it. We strongly argue that the Fe-S machineries are in fact very good additional systems to investigate the placement of organelles.

2) "their components are encoded in the nucleus, avoiding the problem of compositional biases;" This is not the case for SufB which is often plastid encoded in red algae and secondary algae! See for example Janouškovec 2015

We apologize for the oversimplification. Indeed, SufB and SufC are encoded in plastids in Cryptophyta, Glaucophyta, Rhodophyta, Apicomplexa, and Diatoms. We have amended the text accordingly.

3) "being part of a highly integrated process they were likely subjected to similar evolutionary constraints." First of all, the authors should explicit how this would improve phylogenetic resolution. Most importantly, the method section mentions that "Markers that were not broadly distributed (IscX in eukaryotes, SufU and SufT in Cyanobacteria) were eliminated. We also discarded HscB and IscU as they did not form clear monophyletic groups in preliminary trees." This is evidence that the different components of these machines are NOT subjected to similar evolutionary constraints.

We kindly disagree. For the proteins that are not widespread, it means that they were lost, which does not preclude the others to coevolve. For the lack of monophyly in the HscB and IscU single gene trees, the non-monophylies are not supported and are likely due to the lack of signal at such evolutionary depth. The important message is that most components have a consistent signal.

--- The authors underplay the lack of resolution of theirs results ---

The authors first highlight the congruence of their results with what's been inferred from the most sophisticated phylogenomic analyses on the question:

"Bayesian analysis using the CAT+GTR model robustly supports the monophyly of Eukaryotes (PP=1) and their deep branching just after Magnetococcales (PP=0.82), strengthening the Alphaproteobacteria-deep hypothesis4,5" while, in fact, the two cited papers (4,5) recover a more nested position of eukaryotes! Why are the authors not mentioning this incongruence?

We apologize if it was unclear. What we meant was that our analyses support a deep emergence of mitochodria and not with Rickettsiales. We now clarify the fact that we recover a deeper placement prior to the divergence of MP1.

Similarly, for the SUF analyses, they write that "Bayesian and ML analysis supports a deep placement of plastids within Cyanobacteria as also indicated by previous analyses9,10." while in fact the Bayesian phylogeny is almost completely unresolved when it comes to placement of plastids.

We have rerun a new analysis with increased taxonomic sampling, and this is now much more robust. The figure and text have been changed accordingly.

Finally, in that analysis, the different approaches yield three different placements for plastids that are listed without much discussion at all or argument for how to explain this or which is most likely the correct placement.

Given the new and more solid results, these are now clarified in the revised version.

So not only was there no controversy about the placement of plastids in cyanobacteria in the literature before this work, contrary to the framing of the authors, but here the results do not bring any further insights into it, they're simply inconclusive.

Again, we clearly state that we support recent data using an alternative dataset. Our results are certainly not inconclusive.

CONCLUSION:

In conclusion, this work tries to address non-controversial questions with less sophisticated analyses and inconclusive results, while misrepresenting the state-of-the-art and the relevance of their approach. I do not think this is up to the standards of publication in Plos Biology.

We are genuinely surprised by the hostility of this reviewer. We agree that the issue will luckily become less and less discussed, but the debate is still too recent to say it’s already perceived as fully non-controversial in the large scientific community, also considering the last correspondence between the Ettema and Martin groups on NEE, which we cannot simply ignore.

It’s exactly what happened with the 3D versus 2D domain of life, it took a few years and a lot of analyses all going in the same direction to solidify it in the eyes of the scientific community. Here it is the same, we provide additional support to three very recent analyses using the key eukaryotic process of [Fe-S] cluster synthesis, and the more evidence we have the better. Moreover, the value of our analysis is clearly recognized by the other two reviewers.
